# Chat-Messenger-Nutzung in der Parkinson-Versorgung

**DOI:** 10.1007/s00115-024-01686-6

**Published:** 2024-07-22

**Authors:** A. Naegele, I. Schwaninger, L. Toenges, C. Eggers, T. Warnecke, C. Weigand, J. Klucken

**Affiliations:** 1 dmac Medical Valley Digital Health Application Center GmbH, Bamberg, Deutschland; 2 Digital Medicine Group, Luxembourg Centre for Systems Biomedicine (LCSB), University of Luxembourg, Esch-sur-Alzette, Luxemburg; 3 Deutsche Gesellschaft für Parkinson und Bewegungsstörungen, Berlin, Deutschland; 4 Klinik für Neurologie, St. Josef-Hospital, Universitätsklinikum der Ruhr-Universität Bochum, Bochum, Deutschland; 5 Neurodegeneration Research, Protein Research Unit Ruhr (PURE), Ruhr University Bochum, Bochum, Deutschland; 6 Klinik für Neurologie, Universitätsklinikum Marburg, Marburg, Deutschland; 7 Klinik für Neurologie, Knappschaftskrankenhaus Bottrop, Bottrop, Deutschland; 8 Klinik für Neurologie und Neurologische Frührehabilitation, Klinikum Osnabrück, Osnabrück, Deutschland; 9 Parkinson Zentrum Osnabrück, Akademisches Lehrkrankenhaus der Universität Münster, Osnabrück, Deutschland; 10 Fraunhofer IIS Mobile Health Lab, Erlangen, Deutschland; 11 Digital Medicine Group, Luxembourg Institute of Health (LIH), Strassen, Luxemburg; 12 Digital Medicine Group, Centre Hospitalier de Luxembourg (CHL), Luxembourg, Luxemburg

**Keywords:** Arzt-Patient-Kommunikation, Telemedizin, Datenschutz, Digitale Gesundheitstechnologien, Elektronische Patientenakte, Physician-patient communication, Telemedicine, Data protection, Digital healthcare technologies, Electronic patient files

## Abstract

**Hintergrund:**

Die Nachfrage nach Chat-Messengern für den Austausch zwischen Ärzten, Therapeuten und ihren Patienten steigt. Die Erwartungen an die Chat-Messenger und Unsicherheiten hinsichtlich Einführung und Nutzung sind heterogen.

**Ziel der Arbeit:**

Die Implementierung eines Chat-Messengers in die Parkinson-Versorgung soll durch die Formulierung einführungs- und anwendungsrelevanter Empfehlungen vereinfacht werden.

**Methodik:**

Semistrukturierte Interviews mit Neurologen und Physiotherapeuten wurden durchgeführt, um die Erwartungen an die Nutzung von Chat-Messengern zu erfassen, welche die Basis für die abgeleiteten Empfehlungen bildeten.

**Ergebnisse:**

Die Erwartungen an die technische Funktionalität übersteigen die Funktionen von Chat-Messenger. Das betrifft z. B. die Anbindung des Chat-Messengers an die elektronische Patientenakte. Insbesondere auch bei den anzuwendenden Regelungen der Datenschutz-Grundverordnung (DSGVO) herrscht große Unsicherheit. Die Empfehlungen, die sich auf die Nutzung der Chat-Messenger, auf die Aspekte des Datenschutzes, die Gestaltung dieser Technologien und methodische Überlegungen beziehen, sollen bei der Implementierung des Tools als zusätzlichen Kommunikationskanal helfen.

**Diskussion:**

Aus den Ergebnissen leiten sich Empfehlungen zur Funktionalität, dem Umgang mit Chat-Messengern im Alltag und in Bezug zum Datenschutz ab. Durch Verbesserung der Kenntnisse bei den Gesundheitsberuflern und Beachtung dieser Handlungsanweisungen können Ärzte und Therapeuten zu einer erfolgreichen Etablierung des Chat-Messengers als zusätzliches Kommunikationstool beitragen.

**Zusatzmaterial online:**

Die Online-Version dieses Artikels (10.1007/s00115-024-01686-6) enthält die ausführliche Darstellung der Methodik zur Umfrage.

## Abstract (en)

**Background:**

The demand for chat messaging apps for communication between physicians, therapists and patients is increasing. The expectations for this form of communication and uncertainties regarding introduction and use are heterogeneous.

**Objective:**

The implementation of chat messengers in the care of patients with Parkinson’s disease should be facilitated by recommendations regarding introduction and usage.

**Methods:**

Semi-structured interviews with neurologists and physiotherapists were conducted to capture the expectations and needs regarding the use of chat messengers. From the data analysis, recommendations were derived.

**Results:**

The expectations for technical functionality exceeded the chat messenger functions. This concerns, e.g., the connection of the chat messenger to the electronic patient file. There is a great deal of uncertainty, particularly when it comes to the applicable General Data Protection Regulations (GDPR). The recommendations relating to the use of chat messengers, data protection aspects, the design of such tools and methodological considerations can help to implement the tool as an additional communication channel.

**Conclusion:**

Practical recommendations regarding functionality, the use of chat messengers in everyday life and in relation to data protection are derived from the results. By improving knowledge, physicians and therapists can contribute to the successful establishment of chat messengers as an additional communication tool.

## Hintergrund

Die optimale Parkinson-Versorgung erfordert eine gute Kommunikation zwischen Patienten, Ärzten und Therapeuten [12]. Viele „kleine Fragen“ beschäftigen die Patienten im Alltag des chronischen Krankheitsverlaufs. Wie in anderen Bereichen unseres Lebens auch könnten Chat-Messenger die oft ineffiziente zeitliche und örtliche Anwesenheit der Beteiligten bei Telefonaten und Gesprächen in der Praxis umgehen [11]. Auch die Leitliniengruppen empfehlen den supportiven Einsatz digitaler Gesundheitstechnologien in der Diagnosestellung [14]. Leider stehen v. a. Unsicherheiten und Unerfahrung mit der digitalen Transformation der Medizin, die Unkenntnis des Datenschutzes und sehr heterogene Erwartungen an die Nutzung eines Messengers dem Einzug der digitalen Lösung für die Kommunikation mit Patienten entgegen. Unter dem Begriff „Chat-Messenger“ werden verschiedene digitale Dienste mit unterschiedlichsten Funktionskomponenten zusammengefasst, welche die Kommunikation zwischen mehreren Nutzern unterstützen. Chat-Messenger bieten dabei eine kurzfristige Informationsmöglichkeit zwischen mindestens zwei Nutzern, die primär *orts*- und *zeitunabhängig* möglich ist (asynchrone Kommunikation, d. h., Fragen und Antworten können zeitlich versetzt stattfinden). Dies vereinfacht die Organisation der Kommunikation und die Teilnehmer sind bez. der Arbeits- oder Alltagsabläufe flexibel. Dringender Informationsaustausch ist jedoch im Vergleich zu Telefonaten oder den persönlichen Gesprächen vor Ort eingeschränkt. Auch die Vertraulichkeit bzw. die Dokumentationsfähigkeit der ausgetauschten Information unterliegen aufgrund der digitalen Übertragung bzw. der Nutzung der Technologie anderen Bedingungen.

Chat-Messenger-Anwendungen weisen dabei die typischen Standardfunktionen auf: das Versenden von Textnachrichten, (aufgenommenen oder gespeicherten) Bildern und Videos sowie anderen Dokumenten- und Dateitypen. Zusatzfunktionen sind die (Video‑)Telefonie, Gruppenchats oder das Versenden von Emojis.

Die Vertraulichkeit der ausgetauschten Information ist in der Medizin ein hohes Gut: Die „ärztliche Schweigepflicht“ wird von Ärzten und Therapeuten gleichermaßen als selbstverständlich wahrgenommen, ist zudem auch zentraler Teil der medizinischen Ethik, der jeweiligen Berufsordnungen und findet sich auch im Hippokratischen Eid wieder [3]. Auch das allgemeine Privatrecht stützt durch § 630 Bürgerliches Gesetzbuch (BGB) die Nutzung von Messengern als Teil der medizinischen Dokumentationspflicht. Nicht zuletzt durch europäische und nationale Normen der Datenschutz-Grundverordnung (DSGVO) wird dies nicht immer als positiver Wert empfunden, sondern auch als organisatorische Hürde, wenn es um Austausch von Gesundheitsdaten und Personenidentifikationen geht (Art. 5 DSGVO; [1, 7, 11]). Um eine qualitativ hochwertige und ethisch verantwortungsvolle Gesundheitsversorgung praktizieren zu können, ist der Schutz der Gesundheitsdaten eine entscheidende Voraussetzung. Jede Form des (digitalen) medizinischen Austausches muss diesen Anforderungen entsprechen, um die Privatsphäre des Einzelnen zu schützen.

Die Umsetzung des angemessenen Datenschutzes bereitet technische und juristische Probleme

Aktuell bereitet die Umsetzung des angemessenen Datenschutzes im Rahmen der Nutzung von Chat-Messengern jedoch an vielen Stellen technische und juristische Probleme und führt zu mehr Fragen und Ängsten als zu praktikablen Lösungen. In der vorliegenden Pilotstudie wurden mit der Parkinson-Versorgung erfahrene Neurologen und Therapeuten (Physiotherapie, Logopädie) der Arbeitsgruppe Parkinson-Netzwerke e. V. semistandardisiert interviewt, um einen ersten Eindruck von den Erwartungen und Hindernissen an Kommunikationsunterstützung und Funktionalitäten der Chat-Messenger zu bekommen. Dabei wurde eine qualitative Methodik eingeführt, um sowohl die prozessualen Aspekte als auch das grundlegende Verständnis über Chat-Messenger-Funktionen zu erfassen. Aus den Erkenntnissen wurden praxisbezogene Empfehlungen abgeleitet, um die Auswahl und Einführung eines Chat-Messengers als erweiterten Kommunikationskanal zu ihren Patienten einführen zu können.

## Methodik

Das gegenwärtige Verständnis und die Erwartung der Ärzte und Therapeuten an Chat-Messenger wurde mithilfe semistrukturierter Interviews nach Helfferich untersucht [10]. Die Befragungen wurden mittels Videochats durchgeführt und dauerten im Schnitt 40 min. Abgefragt wurden die derzeitigen Nutzungsgewohnheiten von Chat-Messengern, aufgetretene Probleme bei Einführung sowie Vorteile während der Nutzung von Messengern. Der zentrale Fokus der Erhebung lag auf den Hindernissen bei Einführung und den Erwartungen, die die Befragten an Messenger benannten.

Hierzu wurde ein strukturierter Leitfaden für die Interviews entwickelt, der die Aspekte *Nutzung eines Chat-Messengers* und *(potenzielle) Probleme bei Einführung und Nutzung* abdeckte. Eine ausführliche Darstellung der Methodik ist im Onlinezusatzmaterial einzusehen und bei den Autoren zu erfragen.

Insgesamt wurden vier Neurologen, zwei Physiotherapeuten und eine Logopädin interviewt, wobei diese meist noch keine Erfahrung mit Chat-Messenger-Diensten in ihrem Versorgungskontext hatten, jedoch sehr wohl im privaten Bereich. Diese Befragten wurde ausgewählt, um basierend auf deren Kenntnisständen Handlungsempfehlungen formulieren zu können, die beim initialen Implementieren des Chat-Messengers in den Versorgungsprozess helfen können. Die Auswahl der Befragten wurde über die Zugehörigkeit zu Parkinson Netzwerke e. V. gewählt, um Erfahrungen und Motivation zur integrierten/vernetzten Versorgung zu gewährleisten. Die Teilnehmerzahl ist begrenzt und stellt keine repräsentative Auswahl der Berufsgruppen dar.

Die Audiodateien wurden manuell transkribiert und die Inhalte strukturiert ausgewertet. Die Ergebnisse wurden nach den Kategorien *Unsicherheiten bei der Nutzung* sowie *Erwartungen an Chat-Messenger *sortiert. Eine ausführliche Beschreibung des Interviewaufbaus findet sich im Onlinezusatzmaterial dieses Papers. Um die qualitativen Erkenntnisse der Befragungen systematisch zu analysieren, wurde die Methode der Themenanalyse in Anlehnung an die von Braun und Clarke angewandt, um eine gründliche und nachvollziehbare Auswertung zu gewährleisten und fundierte Handlungsempfehlungen abzuleiten [5]. Dieser Ansatz ermöglichte es, Kategorien in den Erhebungen zu identifizieren, die Aufschluss über die Wahrnehmungen und Erfahrungen der Befragten im Kontext der Nutzung von Chat-Messengern gaben. Die befragten Neurologen repräsentieren die Erwartungen der Ärzte, während die Physiotherapeuten stellvertretend für Therapeuten befragt wurden. Die Empfehlungen beziehen sich auf die Gesundheitsberufler selbst, auf die geeignete Patientenauswahl für die Messenger-Kommunikation sowie datenschutzrechtliche Aspekte.

## Ergebnisse

### Unsicherheiten bei der Nutzung eines Messengers

Im ersten Schritt wurden die Aspekte der Einführung von Chat-Messengern sowie deren Nutzung im Versorgungsalltag beleuchtet (Tab. 1). Dabei zeigte sich, dass vornehmlich die Vielzahl an Datenschutzregelungen sowie die unklaren Vergütungsmöglichkeiten dieser zusätzlichen Kommunikationsmöglichkeit für die Patienten Unsicherheiten bei den Befragten darstellen. Zudem wurden auch die körperlichen Voraussetzungen der Patienten hinterfragen und der Verlust essenzieller Bestandteile der Kommunikation mit Patienten (z. B. Feedback, Emotionen) hervorgehoben.

Es ist festzuhalten, dass die meisten Befragten noch keine Praxiserfahrung mit Messengern hatten, da deren Einführung durch diverse Abstimmungshindernisse und Probleme bei der Messenger-Auswahl verhindert wurde. Insbesondere sticht hier die große Unsicherheit hinsichtlich der einzuhaltenden Datenschutzregelungen gemäß der DSGVO hervor. Eine ausführliche Auflistung der aufgeführten Punkte ist im Onlinezusatzmaterial zu finden.

**Tab. 1:** Zusammenfassung der in den Interviews geäußerten Probleme während Einführung und Nutzung eines Messengers

Bereich	Wahrgenommene Probleme und Unsicherheiten seitens der Ärzte und Therapeuten^a^
Arzt, Praxis, Krankenhaus	Vielzahl an zu beachtenden Datenschutzregelungen (4/7)
	Nachteilige Vergütung für Nutzung des Messengers als Zusatzleistung (4/7)
	Lange Abstimmungsprozesse für die Auswahl des Messengers (1/7)
	Mangelnde Ressourcen für die Auswahl des Messengers (1/7)
Patienten, Parkinson	Mangelnde körperliche Voraussetzungen für die Bedienung eines Smartphones und eines Messengers (1/7): Überforderung der Patienten durch Messenger-Mitteilungen (1/7) Unübersichtlichkeit des Messengers für Patienten (1/7) Großteil der Patienten besitzt kein Smartphone (1/7)
	Fehlende Bereitschaft seitens der Patienten für den Download eines weiteren Messengers für die Kommunikation mit dem GB (1/7)
	Unlimitierte Verfügbarkeit des Arztes könnte eine Abhängigkeit des Patienten hervorrufen (1/7)
Kommunikation und Infrastruktur	Leidende Arzt-Patient-Beziehung, falls der Messenger der ausschließliche Kommunikationskanal ist (2/7)
	Fehlendes Feedback, ob der Patient Nutzung richtig verstanden hat (1/7)
	Verlust der Emotionen als wichtigen Teil der Kommunikation (1/7)
	Hemmnisse der Patienten, mittels Messenger intime Probleme zu besprechen (1/7)
	Unzureichende Netzabdeckung in flach-ländlichen Regionen (1/7)

*GB* Gesundheitsberufler

^a^In Klammern: Häufigkeit, mit welcher der jeweilige Punkt von den sieben Betroffenen angesprochen wurde

### Erwartungen an einen Chat-Messenger

Alle Befragten äußerten den klaren Bedarf nach Chat-Messengern als ergänzenden, bidirektionalen Kommunikationskanal mit ihren Parkinson-Patienten (Tab. 2, Onlinezusatzmaterial). Sechs von sieben Befragten haben höhere Erwartungen an den Chat-Messenger als dieser tatsächlich bieten kann. Die repräsentativ befragte Gruppe von Neurologen und Physiotherapeuten hat ein breites Interesse für diverse Themen der Digitalisierung und signalisieren das Bedürfnis nach einer verbesserten sektorenübergreifenden Kommunikation. So soll der Chat-Messenger eine flexible Kommunikation ermöglichen: Mehrere Endgeräte sollen für die Übermittlung diverser Dateiformate genutzt werden können. Auch die inhaltliche Strukturierung von Patientendaten sowie die Integration in die elektronische Patientenakte wurde von manchen der Befragten erwartet.

**Tab. 2:** Zusammenfassung der Erwartungen an Chat-Messenger seitens der Neurologen und Physiotherapeuten

Funktion/Komponente	Erwartungen der Neurologen und Physiotherapeuten^a^
Technische Erwartungen an einen Chat-Messenger	Austausch aller gängigen Nachrichtenformate (Bild, Video etc.; 3/7)
	Austausch in Gruppen (intrakollegial und mit Patienten; 3/7)
	Kommunikation über mehrere Endgeräte (3/7)
	Kommunikation auch mittels Videotelefonie (2/7)
	Eingabe mittels Diktierfunktion und automatischer Texterkennung (1/7)
Inhaltliche Erwartungen an einen Chat-Messenger	Filterung der Kontaktliste (Patientenliste) nach bestimmten Variablen (1/7)
	Ausspielen von informativen Beiträgen an gefilterte Patientenauswahl (1/7)
	Anfertigen von Dokumenten, die die Patienteninformationen (PROMS, PREMS etc.) strukturiert darstellen (1/7)
	Senden von Patientendokumenten und -informationen an die ePA (1/7)
	Manuelle Archivierung für einzelne Chatverläufe (1/7)
	Bereitstellung bzw. Befüllung des Medikationsplans (1/7)
	Ein Physiotherapeut erwartet eine Monitoringfunktion, mit der der Patient die Möglichkeit hat, den eigenen Gesundheitszustand im Verlauf aufzuzeichnen

*ePA *elektronische Patientenakte*, PROMS *„patient reported outcome measures“*, PREMS *„patient-reported experience measures“

^a^In Klammern: Häufigkeit, mit welcher der jeweilige Punkt von den sieben Betroffenen angesprochen wurde

Zusammenfassend ist zu sagen, dass die Bereitschaft für die Integration neuer Kommunikationstools in den medizinischen Alltag unter allen Befragten vorhanden ist. Das Bedürfnis, die generelle Kommunikation im Gesundheitswesen (mit Messengern) auszubauen, ist stark ausgeprägt, da besonders die sektorenübergreifende Informationsweitergabe zwischen allen Beteiligten wiederkehrend problematisch ist. Viele Erwartungen der Gesundheitsberufler können mit den aktuell gängigen Messengern nicht realisiert werden und übersteigen die technischen Möglichkeiten eines Chat-Messengers. Ein fachgruppenspezifischer Unterschied zwischen den genannten Anforderungen der befragten Neurologen und Physiotherapeuten konnte nicht identifiziert werden. Hierfür müssen weiterführende Forschungen mit einer größeren Stichprobe und ausgeglichener Verteilung der Berufsgruppen erfolgen.

## Zusammenfassung und Empfehlungen

Bevor ein Messenger als weiterer optionaler Kommunikationskanal genutzt werden kann, sollte sich der Gesundheitsberufler mit einigen Regelungen und Fragestellungen auseinandersetzen, die die Implementierung eines Messengers in die Patientenversorgung vereinfachen. Die Empfehlungen wurden größtenteils aus den Erwartungen und geschilderten Hindernissen abgeleitet, die im Rahmen der Patientenversorgung bei der Nutzung eines Chat-Messengers geschildert wurden. Auch die Einschätzung, ob sich der Patient für die Kommunikation mit einem Chat-Messenger eignet, liegt initial beim Gesundheitsberufler.

- Etablierung einer realistischen Erwartungshaltung gegenüber Chat-Messengern
- Respektierung des Datenschutzes
- Umstrukturierung des Arbeitsalltags
- Einholung von Informationen über die Möglichkeiten der Vergütung der Leistung. (Hier sind insbesondere der Einheitliche Bewertungsmaßstab [EBM] und die Gebührenordnung für Ärzte [GOÄ] bzw. die DRG[„diagnosis related groups“]-Fallpauschalen zu beachten)
- Prüfung der Eignung des einzelnen Patienten für den Austausch mittels Chat-Messenger

Die aufgeführten Empfehlungen wurden mit den Mitgliedern des Parkinson Netzwerk e. V. diskutiert. Durch die Berücksichtigung dieser Empfehlungen tragen die Gesundheitsberufler dazu bei, entscheidende Voraussetzungen für eine zielführende Implementierung eines Chat-Messengers für die erweiterte Kommunikation zu ihren Patienten zu schaffen.

### Einführung von Chat-Messengern bei passenden Parkinson-Patienten

Neben dem Gesundheitsberufler sollte auch der Parkinson-Patient einige Voraussetzungen erfüllen, die die Nutzung eines Messengers als weiteren Kommunikationskanal ermöglichen. Im Folgenden sind daher Empfehlungen aufgelistet, auf welche die Gesundheitsberufler bei Parkinson-Patienten achten sollten, um deren Tauglichkeit für die Nutzung von Chat-Messengern einschätzen zu können. Dabei handelt es sich um indirekte Ableitungen aus den durchgeführten Interviews mit den Ärzten und Physiotherapeuten gemäß den Empfehlungen des National Institute for Health and Care Excellence [15].

- Entsprechende körperliche und psychomotorische Voraussetzungen für die Bedienung eines Smartphones mit Chat-Messenger
- Bereitschaft des Patienten, über den Chat-Messenger Informationen hinsichtlich seiner Gesundheit auszutauschen
- Sichere Bedienung des Chat-Messengers durch den Patienten
- Notwendiges Verständnis über grundlegende Datenschutzaspekte

Eine entsprechende physische und psychische Verfassung des Patienten sowie eine positive persönliche Einstellung zur digitalen Kommunikation und ein gewisses technisches Wissen sollten vorhanden sein, um die Vorzüge aus der Kommunikation mittels Chat-Messenger auf die Kommunikation zwischen Gesundheitsberufler und Patienten zu übertragen. Dabei dürfen ggf. fehlende Voraussetzungen nicht erzwungen werden, um die Kommunikation mit dem Patienten über dieses Tool starten zu können. Dies hätte negative Folgen für das vertrauensvolle Verhältnis zum Patienten und würde sich nachteilig auf die Messenger-Kommunikation auswirken. Wie auch in der Literatur empfohlen [16], sollten Tools für asynchrone Kommunikation jedenfalls ergänzend zu tatsächlichen Visiten stattfinden und nicht als Ersatz.

### Aspekte des Datenschutz und der Datensicherheit

Während einer Patientenbehandlung oder -therapie werden Gesundheitsdaten (medizinisch relevante Informationen) erfasst, die den Patienten als spezifische Person für Dritte identifizierbar machen und deshalb als sensible Daten verstanden werden [7]. Darunter fallen beispielsweise medizinische Diagnosen, genetische Informationen oder die Angaben zur psychische Gesundheit [6], die als „besonders schützenswerte personenbezogene Daten“ definiert sind [11]. In Deutschland gilt die europäische DSGVO und das Bundesdatenschutzgesetz (BDSG), welches die DSGVO konkretisiert und vervollständigt [1]. Öffentliche Stellen (wie beispielsweise Praxen und Krankenhäuser) müssen sicherstellen, dass diese Daten transparent und rechtmäßig verarbeitet werden (§ 2 BDSG). Die Regelungen der DSGVO und des BDSG gelten natürlich auch für Chat-Messenger. Die wichtigsten Aspekte für die Alltagsnutzung werden im Folgenden dargelegt und in Tab. 3 zusammengefasst.

**Tab. 3:** Empfehlung für den Start der Nutzung des Chat-Messengers

1	Der Arzt hat sich für einen Chat-Messenger-Anbieter entschieden
2	Der Arzt hat eine EWE formuliert, die er bei Bedarf direkt in der Praxis/im Krankenhaus aushändigen kann
3	Der Arzt behandelt einen Patienten, dessen Situation eine ergänzende Kommunikation über den Messenger grundsätzlich erlaubt
4	Der Arzt nennt und erklärt dem Patienten die Möglichkeit der Kommunikation über einen Messenger
5	Der Patient erhält die EWE und willigt in die Nutzung des Messengers ein
6	Der Arzt prüft final die Tauglichkeit des Patienten für die Kommunikation über einen Messenger (siehe „Empfehlungen an die Auswahl geeigneter Parkinson-Patienten“)
7	Der Arzt zeigt dem Patienten den automatisch generierten QR-Code, den der Patient mit seinem Smartphone abscannt und dadurch mit dem Messenger-Account des Arztes verbunden wird
8	Der Arzt händigt dem Patienten ein Informationsblatt aus, welches die Funktionen des Messengers erklärt. Zudem sollte der Arzt im persönlichen Gespräch eine kurze Einführung in das Tool geben, da so am wahrscheinlichsten Verständnisprobleme aufgedeckt und behoben werden können
9	Ab hier startet die Dokumentationspflicht der Inhalte

*EWE* Einwilligungserklärung

Die Chat-Messenger-App bildet den digitalen Vernetzungspunkt zwischen Gesundheitsberufler und Patient. Bei der Auswahl des Chat-Messengers sind daher die technischen Aspekte zu beachten, die dem Datenschutz entsprechen (Tab. 4) und somit eine ethisch und technisch verantwortungsvolle Übermittlung sensibler Patienteninformationen ermöglichen. Neben diversen kommerziellen Anbietern wird derzeit auch von der gematik eine zertifizierte TI-Messenger-App in erster Entwicklungsstufe angeboten.

**Tab. 4:** Zusammenstellung der empfohlenen technischen Aspekte, die ein Chat-Messenger erfüllen sollte, um DSGVO-konform Daten zu verarbeiten und zu übermitteln

„Ende-zu-Ende-Verschlüsselung“ für eine sichere Übertragung aller Daten
Nach erstmaligem Öffnen der Messenger-App müssen die Datenschutzhinweise angezeigt werden und in den Einstellungen der App jederzeit nachlesbar sein
Archivierungsfunktion einzelner Chatverläufe, um der Aufbewahrungsfrist nachkommen zu können
Verfügbarkeit des Messengers auf den gängigen Betriebssystemen iOS und Android
Möglichkeit der Löschung von Daten sowohl einzelner bereits übermittelter Nachrichten als auch des gesamten Accounts des jeweiligen Nutzers
Möglichkeit der Entfremdung von Patientendaten bei intrakollegialem Austausch (z. B. durch Schneide- oder Zeichentools im Messenger)
Der Unternehmenssitz des Messenger-Anbieters sollte innerhalb der EU sein. Die Nutzung des Chat-Messengers unterliegt auf jeden Fall den Vorschriften der DSGVO
Vorgelagerter Zugriffsschutz, um sicherzustellen, dass die Daten lediglich von Autorisierten eingesehen werden können

*DSGVO *Datenschutz-Grundverordnung, *EU* Europäische Union

### Einführung des Chat-Messengers in die individuelle Kommunikation

Bevor ein Austausch über die Chat-Messenger-Plattform stattfinden kann, muss der Patient seine Einwilligung in die Erhebung und Verarbeitung seiner Patientendaten erklären, die während der Nutzung vom Chat-Messenger übermittelt werden (Einwilligungserklärung [EWE] – Art. 6 DSGVO; [8]). Dies gilt nicht nur gegenüber dem Hersteller des Chat-Messengers, sondern natürlich auch für die Kommunikation zwischen Patient und Arzt/Therapeut, die ebenfalls eine entsprechende EWE benötigen (Tab. 5, Onlinezusatzmaterial). Gesundheitsberufler müssen laut DSGVO „Rechenschaft ablegen“ (Art. 5 DSGVO) können, es ist ratsam diese schriftlich abzuhandeln. Die EWE ist an keine spezielle Form gebunden [8] und sollte auch Aspekte der Nutzerfreundlichkeit („usable privacy“) einschließen [15].

**Tab. 5:** Empfehlungen für eine vom Arzt einzuholende Einwilligungserklärung vom Patienten (gem. DSGVO)

1. Die EWE muss vom Patienten freiwillig und unmissverständlich für den konkreten Zweck abgelegt werden
2. Sowohl die Einwilligung als auch deren Widerruf müssen für den Patienten einfach umsetzbar sein (Art. 7 DSGVO)
3. Die EWE weist auf die gesetzlich vorgeschriebene Aufbewahrungsfrist von 10 Jahren hin [2]
4. Die EWE definiert, welche Daten wie zwischen Arzt und Patient ausgetauscht werden [8] Angaben zum verwendeten Messenger, Anleitung für den Download des Messengers Definition der Inhalte, die über den Messenger übermittelt werden (dürfen): – Übermittelbare Inhalte (Beispiele): Terminmanagement; Abfrage des aktuellen Befindens; Auftreten von Nebenwirkungen; (körperliche) Probleme im Alltag, die die Parkinson-Krankheit hervorbringt – Nicht zu übermittelnde Inhalte (Empfehlungen): Testergebnisse, die einer näheren Erläuterung bedürfen; Medikationsplan (dieser sollte in persönlicher Absprache vor Ort erfolgen, da so eine geringere Gefahr einer Verwechslung des jeweiligen Plans bzw. des Patienten besteht)
5. Definition der zeitlichen Verfügbarkeit des Arztes
6. Hinweis auf Nichtnutzung des Messengers bei Meldung von Notfällen
7. Nutzerfreundlichkeit bei der Übermittlung der Informationen zur DGSVO und Einwilligungserklärung

*DSGVO *Datenschutz-Grundverordnung, *EWE* Einwilligungserklärung

Wichtig hierbei ist, dass der (organisatorische) Umgang mit diesen Daten dem medizinischen Personal bewusst ist. „Kritische“ Situationen, in denen die Patientendaten in ihrer Vertraulichkeit gefährdet sein könnten – wie beispielsweise nicht zugriffsgeschützte Smartphones mit Patientendaten oder Nichteinhaltung des Datengeheimnisses – sollten bekannt sein und auch dem Patienten erklärt werden [4]. Der Zugriff auf die Patienten-Chatverläufe muss strikt kontrolliert werden, sodass nur die Berechtigten die Chatverläufe einsehen können.

Die initiale Kommunikation mit dem Patienten sollte immer der Gesundheitsberufler eröffnen

Neben der datenschutzrechtlichen Einwilligung und der Kenntnis über die sensiblen Aspekte des Datenaustauschs sollten auch die Rahmenbedingungen der Kommunikation zwischen Patient und Arzt/Therapeut vor der Nutzung des Chat-Messengers abgestimmt werden. Sowohl aus datenschutzrechtlichen Gründen als auch für die Gewissheit, die Kommunikation tatsächlich mit der gewünschten Person zu betreiben, sollte der Gesundheitsberufler die initiale Kommunikation mit dem Patienten eröffnen. So kann der Arzt die Kommunikation zu seinen Patienten steuern und den Patienten geordnet anschreiben. Dabei sollten auch Zweifel und falsche Erwartungen abgestimmt werden. Speziell die Themen *Erreichbarkeit* und *Antwortverzögerung* müssen besprochen werden sowie eine alternative Kontaktaufnahme über Telefon/Notfallnummern in Notsituationen. Besonders bei Nutzern höheren Alters kann dieser Ablauf zur besseren Akzeptanz der digital gestützten Kommunikation beitragen.

### Empfehlungen für die Gestaltung von Chat-Messengern

Aufgrund der genannten Bedürfnisse von Personen mit Parkinson-Krankheit, die im Verlauf der Krankheit höchst divers ausgeprägt sein können, ist zu empfehlen, Chat-Messenger unbedingt angepasst an die Bedürfnisse dieser anspruchsvollen Zielgruppe zu gestalten (vgl. z. B. [16]). Um die Vielfalt der Bedürfnisse dieser Zielgruppe in Bezug auf Chat-Messenger zu verstehen, sind weitere Forschung und Entwicklung notwendig. Anforderungen an Parkinson-Patienten können eine Bandbreite beinhalten und sollten auch die oben genannten physischen und psychischen Bedürfnisse dieser Zielgruppe sowie das technische Verständnis berücksichtigen. So wurde in der Literatur für Patienten der Krankheit Parkinson, einer progredienten neurodegenerativen Erkrankung, die textbasierte asynchrone Kommunikation als wegweisend definiert [16], wobei in anderen Fällen auch Spracheingabe oder Gestik sinnvolle Kommunikationsmodalitäten sein könnten oder eine Kombination mehrerer Modalitäten. Gestaltungsspielraum gibt es zudem in der Art des Versendens oder Erhaltens von Nachrichten, wie illustriert in [16]. Die vorliegende qualitative Pilotstudie sollte durch größere, repräsentative Studien ergänzt werden, die auch die berufsgruppen-spezifischen Aspekte beleuchten, und in Einklang mit der Patientenperspektive bringen. Die eingeführte semistrukturierte Interview-Methodik kann hilfreich sein, Chat-Messenger in die Versorgung patientengerecht zu integrieren.

## Fazit für die Praxis

- Ärzte und Therapeuten haben viele Unsicherheiten in den Bezug auf den Chat-Messenger und eine heterogene Erwartungshaltung, was sich gegen eine zügige Implementierung in den Versorgungsprozess stellt.
- Die meisten Gesundheitsberufler haben zu hohe Anforderungen an die Tools und erwarten beispielsweise die Anbindung an die elektronische Patientenakte, was den Bedarf einer verbesserten (sektorenübergreifenden) Kommunikation hervorhebt.
- Für eine gewinnbringende Implementierung sollten die Gesundheitsberufler u. a. eine realistische Erwartungshaltung an Chat-Messenger etablieren und selbstständig Informationen über die individuellen Vergütungsmöglichkeiten dieser Leistung einholen.
- Um zu prüfen, ob sich der Parkinson-Patient für die Kommunikation mittels Chat-Messenger eignet, müssen die Gesundheitsberufler körperliche und geistige Aspekte beim Patienten sowie dessen Einstellung gegenüber dem Kommunikationsweg berücksichtigen.
- Da die Patientendaten u. a. gemäß des hippokratischen Eides geschützt werden müssen, muss der Datenschutz durchwegs berücksichtigt werden.

### Supplementary Information

Darstellung der Methodik
