# Supplementary material for: Chat messenger use in the care of patients with Parkinson’s disease
Source: Nervenarzt. 2024 Jul 22;95(8):714–20. [Article in German] doi: 10.1007/s00115-024-01686-6 (PMC11297055; doi:10.1007/s00115-024-01686-6)
Supplement: Supplementary file 1 — Darstellung der Methodik [file 115_2024_1686_MOESM1_ESM.pdf]

Online-Zusatzmaterial

Inhalt

Methodik 1

Beschreibung der Befragung 1

Interview Leitfaden zum Thema „Nutzung von Messengern in der Parkinson-Versorgung“ im Detail 2

Literaturverzeichnis 4

Methodik

Beschreibung der Befragung

Die Interviews wurden durchweg über die Video-Chatplattform Microsoft Teams abgehalten, dauerten durchschnittlich 40 Minuten und waren wie folgt aufgebaut: nach einer kurzen Vorstellung der Interviewenden Alina Nägele selbst sowie eines kurzen Einblicks in den Beweggrund für die Interviewreihe wurden ein paar einleitende Fragen an den Interviewten gestellt, die Aufklärung darüber geben sollten, welchen Kenntnisstand der Befragte über einen Messenger hat. An dieser Stelle erfolgte die Abgrenzung eines Messengers zu einem komplexeren Kommunikationstool, um den Befragten eine einheitliche Einschätzung über den möglichen Funktionsbereich des Messengers zu geben. Im weiteren Verlauf folgten Fragen über die aktuelle berufliche Nutzung eines Messengers für die Kommunikation mit den Patienten, die möglichen Probleme bei der Einführung und Nutzung sowie die möglichen Inhalte, die mittels Chat-Messenger mit dem Patienten ausgetauscht werden könnten.

Alle Gespräche wurden aufgezeichnet, transkribiert und den jeweiligen Teilnehmern per Mail zugeschickt. Die Ergebnisse wurden mittels Excel Tabelle ausgewertet und nach Häufigkeit der Nennung der einzelnen Aspekte in nachfolgendem Kapitel beschrieben und interpretiert/eingeordnet.
Abschließend gilt zu betonen, dass bei den Ergebnissen der Interviewreihe der besondere, eher technikaffine Hintergrund der Befragten berücksichtigt werden muss. Die Antwortrichtungen würden vermutlich signifikant variieren, wenn Mediziner ohne die genannten Interessenshintergründe befragt worden wären.

Interview Leitfaden zum Thema „Nutzung von Messengern in der Parkinson-Versorgung“ im Detail

Einführung:

- Vorstellung der Interviewerin (Universität, Studiengang, Empfehlungspaper wird im Rahmen der Masterarbeit als Teilleistung erstellt)
- Hinweis: Im Folgenden soll der **Messenger als ergänzende Kommunikationsmöglichkeit** für Parkinson-Patienten und Ärzte verstanden werden

- Wofür wird die Umfrage durchgeführt?
    - Status quo der Nutzung von Messengern bei der Arzt-Parkinson-Patient-Kommunikation
    - Wo liegen Probleme bei der Umsetzung und Nutzung von Messengern?
    - Erwartungen an die Funktionen eines Messengers?

- Worum wird es im Interview u.a. gehen?
    - Status quo der Nutzung bzw. Meinung zur Nutzung eines Messengers im Arbeitsalltag
    - Erwartete Funktionen eines Messengers

- Testfrage: Was genau ist ein Messenger?
- Erklärung, um denselben Kenntnisstand unter allen Befragten sicherzustellen: Differenzierung zwischen komplexen Kommunikationstool und "reinem" Messenger
    - Messenger-Services:
        - Übermittelbare Inhalte: Text, Fotos, Dokumente, Videos
        - Asynchrone Kommunikation am mobilen Endgerät: Zeit und Ort der Kommunikationspartner müssen nicht identisch sein
        - Gängige zusätzliche Funktionen: Videochat
    - Nicht-Messenger-Services (Integration in komplexeres Kommunikationstool notwendig):
        - Speicherung und Zugriff auf medizinischen Daten über den Patienten, z.B. MRT-Bilder
        - Zugriff und Schreibrechte auf die digitale Patientenakte
        - Tracken mithilfe von Wearables
        - Anbindung an die digitale Patientenakte
- Hinweis Aufzeichnung: Gespräch aufzeichnen, Transkribieren, Danach zuschicken

Warm up:

- Vorstellung des Befragten selbst
- Wie treten Sie **aktuell** mit Ihren Parkinson Patienten in Kontakt?
- Findet **außerhalb der regelmäßigen Kontroll-Termine** mit Ihren Patienten ein Kontakt statt?
    - Z.B. Telefon, Mail, Messenger?

Status quo der Nutzung bzw. Meinung zur Nutzung eines Messengers im Arbeitsalltag/Integration im Arbeitsablauf des Arztes bzw. Therapeuten:

- Nutzen Sie aktuell einen Messenger für die Übermittlung von medizinischen (Patienten-) Daten?
    - Mit Patienten? Kollegen? KK? Versicherungen?
    - **Wenn NEIN**: Wie ist die Übermittlung von Patientendaten aktuell bei Ihnen gelöst worden?
- Erachten Sie es generell als **sinnvoll**, einen Messenger als Ergänzung zur Kommunikation mit dem Patienten zu verwenden? --> Würde Ihnen ein Messenger für die Interaktion mit Ihren Patienten helfen?
    - Wenn ja, warum?
- Was ist Ihnen bei einem guten Messenger **wichtig**? (Ich schreibe parallel mit, da ich mich gern gegebenenfalls am Ende des Interviews nochmals darauf beziehen möchte.)
    - Falls Hilfe nötig: Funktionen? Datenschutz?

Legale Nutzbarkeit/Probleme bei der Einführung & Nutzung:

- Stellen Sie sich bitte vor: Sie wollen einen Messenger bei Ihnen in der Klinik/Praxis einführen.
- Welche Probleme sehen Sie a) bei der **Einführung** und b) bei der **Nutzung** einer solchen Messenger App?
    - z.B. weniger persönlich?
    - Fehlende Unterstützungsmöglichkeiten, wenn nur online?
    - Hinderlich beim Aufbau einer Arzt-Patienten-Beziehung?

- ODER: Welche Probleme **hindern Sie aktuell daran**, einen Messenger Dienst **einzuführen**?
    - z.B. Fülle an Optionen verschiedener Messenger
    - Strikte Datenschutzregelungen
    - IT-Infrastruktur
    - Mangelnde Akzeptanz sowohl seitens Arzt als auch Patienten
    - Frage der Haftung
    - Compliance des Patienten (Know-how?)

Inhalte der Kommunikation:

Inhalte, die nur über einen Messenger transportiert werden oder Inhalte, die ein komplexeres Kommunikationstool erfordern.

- Welche Themen besprechen Sie in einem Regeluntersuchungstermin mit Ihrem Parkinsonpatienten?
    - z.B. Allgemeines Befinden?
    - Symptome?
    - (Umstellung des) Medikationsplans?
    - Körperliche Tests

- Welche Teile des bislang persönlich durchgeführten Patientengesprächs können <u>nicht</u> über einen Messenger thematisiert werden und erfordern das persönliche Erscheinen des Patienten?
- Welche Inhalte erfordern dagegen keine Präsenz seitens des Patienten?

- Wie viel Zeit liegt bei Ihnen i.d.R. zwischen den Untersuchungsterminen eines einzelnen Patienten?
- Gibt es Ihrer Meinung nach Informationen, die "zwischendurch" wichtig wären zu erfahren?
    - z.B. Verunsicherungen des Patienten
    - Zustandsveränderungen

Schluss:

- Zusammenfassung der Anforderungen an einen Messenger
    - Welche Anforderungen an einen Messenger hat sich der Befragte gewünscht?

    --> Einordnen, welche Anforderungen über einen reinen Messenger-Service "machbar" sind und welche nicht

- "Lessons-Learned" Frage an den Interviewten durch dieses Interview
    - Haben Sie das Gefühl, dass das Verständnis von digitalen Services in Messenger- und Nicht-Messenger-Services Ihnen geholfen hat, um zu verstehen, wie Digitalisierung betrieben werden muss?

Literaturverzeichnis

1. www.aerzteblatt.de/nachrichten/120885/Videosprechstunden-haben-sich-etabliert. Zugegriffen am 05. Dezember 2023
2. https://app-help.ginlo.net/docs/de/android/index.html#kontakt-scannen. Zugegriffen am 29. August 2023
3. Barayev, E., & Shental, O. e. (2021). WhatsApp Tele-Medicine - usage patterns and physisians views on the plattform. Israel Journal of Health Policy Research, 9.
4. https://www.bitkom.org/sites/default/files/2020-08/bitkom-prasentation-senioren-in-der-digitalen-welt-18-08-2020.pdf. Zugegriffen am 20. Dezember 2023
5. https://www.blaek.de/arzt-und-recht/aufbewahrung. Zugegriffen am 29. September 2023
6. https://www.bsi.bund.de/SharedDocs/Downloads/DE/BSI/Publikationen/Broschueren/Cyber-Sicherheit_als_Wettbewerbsvorteil.pdf?__blob=publicationFile&v=3. Zugegriffen am 01. Oktober 2023
7. https://www.bundesgesundheitsministerium.de/elektronische-patientenakte.html. Zugegriffen am 29. Dezember 2023
8. https://www.bundesaerztekammer.de/fileadmin/user_upload/BAEK/Themen/Internationales/Bundesaerztekammer_Deklaration_von_Genf_04.pdf. Zugegriffen am 15. August 2023
9. https://diga.bfarm.de/de/verzeichnis. Zugegriffen am 30. Dezember 2023

10. https://digitaltag.eu/sites/default/files/2021-06/210608_DT21_PPT_PK_Digitaltag%20V3_Website_DFA_0.pdf. Zugegriffen am 03. September 2023
11. https://dsgvo-gesetz.de/themen/einwilligung. Zugegriffen am 18. August 2023
12. Eggers, C., Wellach, I., & al., e. (2021). Versorgung von Parkinson-Patienten in Deutschland: Status quo und Perspektiven im Spiegel des digitalen Wandels. Der Nervenarzt, 602 - 610. Von https://link.springer.com/article/10.1007/s00115-020-01027-3
13. https://www.ericsson.com/en/reports-and-papers/mobility-report/mobility-visualizer. Zugegriffen am 15. August 2023
14. https://faq.whatsapp.com/794229125227200/?cms_platform=web. Zugegriffen am 02. August 2023
15. https://www.gematik.de/anwendungen/e-patientenakte. Zugegriffen am 14. November 2023
16. https://www.handelsblatt.com/dpa/messenger-neues-channels-feature-whatsapp-oeffnet-die-info-kanaele/29391756.html. Zugegriffen am 01. Oktober 2023
17. https://www.kbv.de/html/videosprechstunde.php. Zugegriffen am 16. August 2023
18. https://www.kbv.de/html/epa.php. Zugegriffen am 15. März 2023
19. https://www.kbv.de/media/sp/EBM_Gesamt_-_Stand_1._Quartal_2023.pdf. Zugegriffen am 6. August 2023
20. https://www.kbv.de/media/sp/Anlage_31b_Videosprechstunde.pdf. Zugegriffen am 02. August 2023
21. https://www.kbv.de/media/sp/liste_zertifizierte-Videodienstanbieter.pdf. Zugegriffen am 12. Dezember 2023
22. Shabbir, R., & Naveed, S. e. (Juli - September 2023). Evaluation of the Factors behind the Failure of Project Management Practices. Journal of Development and Social Sciences, 12.
23. https://www.sueddeutsche.de/digital/smartphones-fuer-senioren-opa-lernt-jetzt-whatsapp-1.301328. Zugegriffen am 02. Augst 2023
